# Self-organization of bacterial communities against environmental pH variation: Controlled chemotactic motility arranges cell population structures in biofilms

**DOI:** 10.1371/journal.pone.0173195

**Published:** 2017-03-02

**Authors:** Sohei Tasaki, Madoka Nakayama, Wataru Shoji

**Affiliations:** 1 Frontier Research Institute for Interdisciplinary Sciences (FRIS), Tohoku University, Sendai, Japan; 2 Graduate School of Science, Tohoku University, Sendai, Japan; 3 Sendai National College of Technology, Natori, Japan; 4 Institute of Development, Aging and Cancer, Tohoku University, Sendai, Japan; University of Illinois at Urbana-Champaign, UNITED STATES

## Abstract

As with many living organisms, bacteria often live on the surface of solids, such as foods, organisms, buildings and soil. Compared with dispersive behavior in liquid, bacteria on surface environment exhibit significantly restricted mobility. They have access to only limited resources and cannot be liberated from the changing environment. Accordingly, appropriate collective strategies are necessarily required for long-term growth and survival. However, in spite of our deepening knowledge of the structure and characteristics of individual cells, strategic self-organizing dynamics of their community is poorly understood and therefore not yet predictable. Here, we report a morphological change in *Bacillus subtilis* biofilms due to environmental pH variations, and present a mathematical model for the macroscopic spatio-temporal dynamics. We show that an environmental pH shift transforms colony morphology on hard agar media from notched ‘volcano-like’ to round and front-elevated ‘crater-like’. We discover that a pH-dependent dose-response relationship between nutritional resource level and quantitative bacterial motility at the population level plays a central role in the mechanism of the spatio-temporal cell population structure design in biofilms.

## Introduction

Microorganisms form various shapes of colonies on solid surfaces to perform collective activities in the natural and human environment. The colony morphology is determined by the microbial strains and the environmental conditions [1, 2]. Different species of microorganisms produce different colony morphologies in the same environment. For instance, the differences appear in the two-dimensional form, cross-sectional profile (elevation), surface roughness, color, and so on. Scientists have utilized such macroscopic features to identify microbial species [3]. On the other hand, even when we incubate only one microbial strain, numerous types of colonies arise in response to environmental variation, including biofilm structures resistant to environmental stress and antibiotics. These facts imply that the morphology of microbial colony should embody characteristic growth strategies selected by each microbial strain, which may hide new effective ways to control population structures in microbial societies [4–7].

*Bacillus subtilis*, one of the most well-studied bacteria, also exhibits diverse colony patterns on the surface of nutrient agar media, which selectively develop in response to environmental conditions. Among others, nutrition (e.g., peptone concentration) and agar concentration are known as principal environmental factors for the pattern selection [8–14]. In the early 1990’s, *B*. *subtilis* strain OG-01 was shown to generate five distinct patterns: diffusion-limited aggregation (DLA)-like, Eden-like, concentric ring-like, disk-like, and dense branching morphology (DBM)-like, depending on nutrition and agar concentration parameters [12]. On hard agar media, such as containing 1% agar, a dendritic DLA-like pattern arises in low nutrition, while a round Eden-like pattern does in high nutrition. Both types of colonies grow slowly, and take 1–4 weeks for expanding the diameter to 50 mm. On soft media, containing less than 0.8% agar, disk-like, concentric ring-like, and DBM-like patterns arise according to their optimum nutrient levels [12]. These colonies grow rapidly and require 12–24 hours for expanding the diameter to 50 mm. The difference in the expansion rate between these colony patterns was suggested to rely on the cell motility, because a nonmotile mutant with no flagella was shown to form only slowly growing DLA-like or Eden-like patterns under any condition [9]. The other principal factor, nutrition, controls the cell proliferation, and probably the motility on the soft agar. The cellular responses were thought to contribute production of multiple types of colony patterns from this single *Bacillus* strain.

Although a variety of factors can be considered for growing environment, our knowledge is still limited, other than the above two factors. For example, surface-moisture was shown to affect some types of colony patterns [7], and incubation temperature was reported to change the cycle of periodic growth patterns [9]. Alkaline and acidic environment has an adverse effect and generally reduces the size of bacterial biofilms [15–17], while a certain acidic condition was reported to facilitate biofilm formation without affecting growing rate in virulent *Streptococcus* strains [18]. In this study, we report the influence of pH perturbation on biofilm morphology of *B*. *subtilis* strain OG-01. We found two types of biofilms that arise differently according to small pH alteration, and explored their self-organizing process from an experimental assay and numerical simulations. Nutrient resource-controlled motility was suggested to be a key mechanism that divides the two colony patterns in certain conditions, and our model simulation robustly reproduced experimental patterns by corresponding arbitrary parameters.

Resource-controlled motility is widely seen in natural organisms, and has caught attention of scientists that investigate collective migrating behavior of eukaryotes cells [19–22]. Our model has a similarity to the self-generated gradient that is assumed to guide long-range migration of cell collectives in eukaryotes [23], and would be utilized among a wide variety of living organisms for producing various morphological structures.

## Results

### Biofilm morphology varies in response to small environmental pH changes

**Fig 1:**
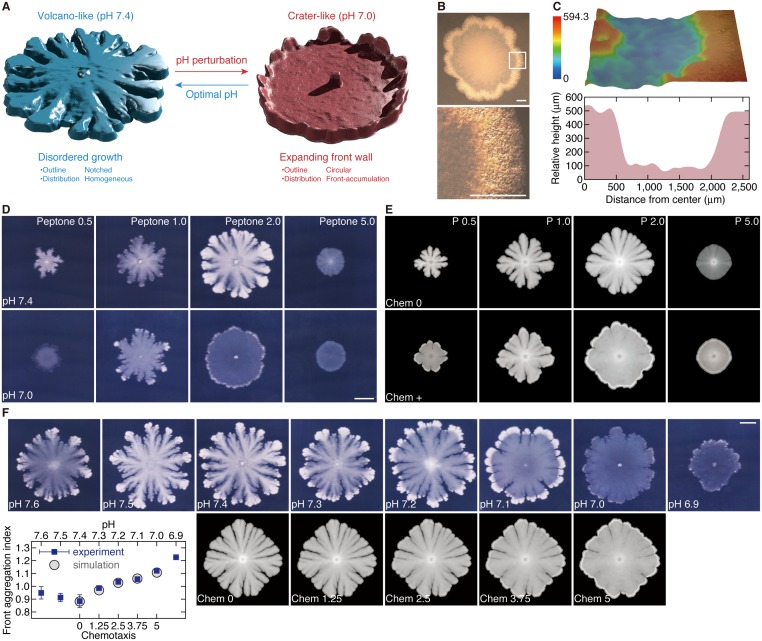
Self-organization of bacterial communities against environmental variations. (A) Colony pattern alteration by a slight pH shift. Shown are three-dimensional views of volcano-like (pH 7.4) and crater-like (pH 7.0) colonies at 5 days post-inoculation, constructed from scanned images. (B) Crater-like colony at 3 days. Boxed region is magnified in the lower panel. Scale bars, 1 mm. (C) Relative height profile of the crater-like colony at 2 days, indicating the bacterial population distribution. The right rim region is elevated by about 400 μm than the inner lower portion. The left center is the inoculation point. Initial nutrition level, 2.0 g l^-1^ peptone (A-C). (D) Colonies under different environmental conditions at 5 days. Initial nutrition level, 0.5, 1.0, 2.0, 5.0 g l^-1^ peptone. pH 7.4 (upper), 7.0 (lower). Volcano-like (pH 7.4) and crater-like (pH 7.0) patterns arise at 2.0 g l^-1^ peptone. Scale bar, 10 mm. (E) Simulated colony patterns. Combination of two parameters, initial nutrition level *P* and chemotactic strength *m*_c_ toward nutrition, illustrates a series of patterns, which are comparable to experimental colonies. Initial nutrition level, *P* = 0.5, 1.0, 2.0, 5.0. Chemotactic coefficients, *m*_c_ = 0 (upper; Chem 0), *m*_c_ > 0 (lower; Chem +). (F) Continuous pH dependence of colony morphology. Representative colonies at 7 days are shown (upper panels). The front aggregation index, an index of cell aggregation at colony boundaries (lower left graph; see S1 Text for details), increases as the pH decreases, at least in the range from pH 7.4 to 7.0. Data represent mean ± s.e.m., *n* = 6. The continuous morphological transition can be simulated by changing chemotactic motility from *m*_c_ = 0 to 5 × 10^−4^: ‘Chem *x*’ means *m*_c_ = *x* × 10^−4^ (lower panels). Blank regions need more elements so far not incorporated into the simulation model. Scale bar, 10 mm.

Biofilms of *B*. *subtilis* on hard agar media are usually classified into two categories by their outline: dendritic patterns (called diffusion-limited aggregation (DLA)-like [24, 25]) and round patterns with coarse interfaces (called Eden-like [26]). The former pattern arises in low nutrition level and the latter in high, and both can be obtained by the inoculation of endospores, resting-phase bacteria. We found that the inoculation of vegetative cells, growing-phase bacteria, exhibit a different colony pattern spectrum. The most characteristic colonies are in the shape of a volcano and a crater, which appear respectively at about pH 7.4 and 7.0 (Fig 1A–1D). The notched outline of the ‘volcano-like’ pattern at pH 7.4 reminisces intermingled features of the previously reported dendritic and round colonies, though its expansion is much faster than both of them. The ‘crater-like’ colony at pH 7.0 has a circular outline and an inhomogeneous bacterial distribution: Its expanding margin is elevated like a crater rim, and its lower inner area is flattened like a basin. Although ‘crateriform’ morphology (a ‘raised’ profile with a small depression) is widely known as an index to identify microbial species by the macroscopic cross sectional shape of the colony, the crater-like pattern, which has a localized rim and a wide flat basin (a large hollow area), has not been reported before.

This morphological change seems to be sudden but is continuous at least in the small range between pH 7.4 and 7.0 (Fig 1F). Because the intracellular pH of *B*. *subtilis* is maintained as around 7.4 within an external pH range [27], a natural question is the effect of an alkaline shift from pH 7.4. At this stage we cannot present a certain characteristics in colony morphology under alkaline conditions over pH 7.4, although a crater-like feature may appear by a small alkaline shift from pH 7.4 similarly to the acidic shift. Morphological response to strongly acidic and alkaline environments is also poorly understood, and requires a deeper understanding of a wider range of microbial activities.

Nutrition, peptone concentration, also greatly affects the colony organization not only in the morphology but also in the expanding rate. The growth radius exhibits a non-monotonic dependence on nutrition level: maximized at an intermediate peptone concentration (~ 2.0 g l^-1^ peptone, forming volcano-like and crater-like patterns), and smaller at lower and higher (Fig 1D). These observations suggest that the volcano-like and the crater-like colonies have new elements in the growth mechanism which have not been observed in the previously discovered colony patterns.

### Directional cell motility is highly sensitive to small pH fluctuation

We investigated what bacterial activities underlie the morphological alteration by the slight pH variation. This range of pH variation does not affect proliferation and sporulation of *B*. *subtilis* [28–31]. In contrast, cell motility may be implicated in the morphological alteration. The effect of external pH variation on bacterial motility has been extensively studied at both single cell and population level [27, 32–37]. For instance, a dramatic motility change was reported around pH 7.5 in a *Streptococcus* strain that lacks an endogenous energy supply, and relatively moderate changes were observed in some bacterial species such as *Escherichia coli* [32, 33, 37] and *B*. *subtilis* [27, 34]. However, the influence of small pH changes have not been examined much.

**Fig 2:**
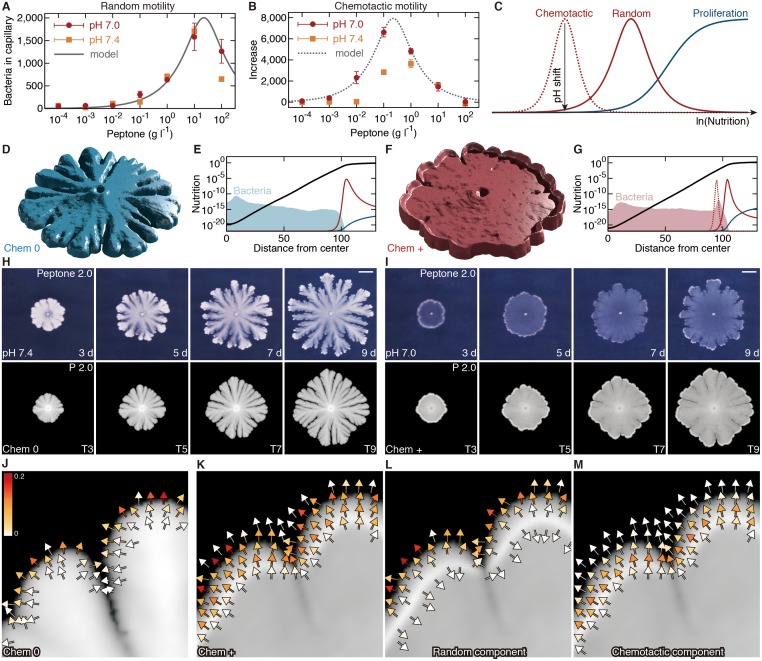
Colony growth simulation designed by resource-response diagram. (A, B) Bacterial motility is quantitatively assessed with a capillary assay. The vertical axis indicates the number of bacteria that move into ‘capillary’, and the horizontal axis the environmental nutrition level, namely the peptone concentration of the surrounding ‘pond’ solution. Random motility is estimated if the capillary is filled with the same solution as in the pond (A). In most cases, a larger number of bacteria are detected if the capillary solution is more nutritious. The increase represents the chemotactic motility coefficient for a fixed nutritional gradient (B). Solid and dotted lines, random and chemotactic motility response curves fitted by our model. Data represent mean ± s.e.m., *n* = 4 (A, B). (C) Schematic diagram of the correlation between nutritional resource level and bacterial response. The vertical axis indicates relative values in each component: random motility, chemotactic motility and proliferation rate. (D, F) Three-dimensional views of simulated colonies. (E, G) Simulated nutrition curves (bold black curves). Shown together are the bacterial concentration *B* and quantitative responses corresponding to the resource-response diagram (color curves) (C). (H, I) Experimental (upper) and simulated (lower) colony growth. Scale bars, 10 mm. (J-M) Vector fields of simulated moving velocity ***u***, indicated by arrows with heat color scale. Moving velocity in the crater-like colony (K) can be decomposed into its random (L) and chemotactic (M) components. Initial nutrition level, 2.0 g l^-1^ peptone (H, I upper); *P* = 2.0 (H, I lower; D-G, J, K). Chemotactic coefficients, Chem 0 for pH 7.4 (D, E, H, J), Chem + for pH 7.0 (F, G, I, K-M). Incubation time, 3, 5, 7, 9 days post-inoculation (H, I upper); *t* = T3, T5, T7, T9 (T*m* = 220*m) (H, I lower); *t* = T5 in D-G, J-M.

We then quantitatively assessed the motility of *B*. *subtilis* with a capillary assay [34, 38–41]. In this assay, two motile properties can be measured at the population level [33]. One is cell motility (undirected, random motility), measured by diffusive behavior in homogeneous environment. The other is chemotaxis (directed, chemotactic motility), an ability to move toward attractants or away from repellents [41]. The result of the assay indicated that little difference in undirected random motility was seen between pH 7.4 and 7.0 (Fig 2A). As for directed movement, the bacteria showed a chemotactic response toward peptone. Higher chemotactic motility was found in pH 7.0 media, compared with in pH 7.4 (Fig 2B). Thus the chemotactic motility toward nutrition can be assumed to change colony morphology from volcano-like (pH 7.4) to crater-like (pH 7.0). The result also suggests that we can selectively and continuously regulate bacterial chemotaxis by making a slight pH adjustment. In contrast to the pH-sensitivity, we found environmental nutrition level has a characteristic effect on both random and chemotactic motility (Fig 2A and 2B). The motility increases until a peak nutrition concentration, and shows an inverse correlation after the transition phase: lower motility in higher nutrition. This motility response indicates that the bacteria actively migrate from nutrient-poor environment to a better place to live whereas almost immotile in sufficiently nutrient-rich environment. Below a critical starvation level, they may abandon migration to hibernate to survive. The non-monotonic motility response to nutrient levels appears to be reflected in the colony radius (small at high and low nutrients) at different initial nutrients in our experiments, and can be fitted by inverted U-shaped dose-response curves, such as:

$$m{\left(\frac{N}{\kappa +N^{2}}\right)}^{\alpha }$$

where *N* is the nutrition level (Fig 2A and 2B). The coefficients *m*, *κ* and *α* denote the motility constant, the peak-motility nutrition squared constant, and the nutrition-response sharpness constant, respectively (S1 Text).

A similar motility response can be expected even on surface environment, although the coefficients probably have different values from those in the motility assay performed in liquid environment where oxygen is poor and nutrition is relatively spatially homogeneous and keeps the initial level. In colony growth on surface environment, in particular, nutrition level is assumed to be much lower than the assigned initial peptone concentration because of consumption by bacteria, and therefore, nutrition-poor behavior is presumed to play a major role in cell population organization on surface environment. For this reason, and for simplicity, we will associate the pH shift with the presence of chemotaxis toward nutrition as seen in a low nutrition region in Fig 2B.

### Population scale model

On the basis of the above bacterial properties, we constructed a mathematical model of two-dimensional colony growth on the surface of solid media (see S1 Text for details). The model is described by the nutrition level *N* = *N*(***x***, *t*) and the bacterial concentration *B* = *B*(***x***, *t*), where (***x***, *t*) denotes the space and time variables. The basic framework follows standard differential equations [42–45], but we reflect the motility response (Fig 2A–2C) to the bacterial velocity ***u*** = ***u***(*N*, *B*) as:

$$u(N,B)=-m_{\text{r}}{\left(\frac{N}{\kappa_{\text{r}}+N^{2}}\right)}^{\alpha_{\text{r}}}\Theta \nabla B+m_{\text{c}}{\left(\frac{N}{\kappa_{\text{c}}+N^{2}}\right)}^{\alpha_{\text{c}}}\nabla N$$

The first term is a random component that approximately expresses various types of diffusional mobility such as undirected random flagellar motility, sliding by proliferating pressure of the bacteria themselves, and Brownian thermal motion. Here, Θ is a random matrix that gives fluctuation. The second term denotes chemotactic motility toward nutrition, defined as directed movement along the spatial gradient of *N*. Both components are regulated by the nutritional dose-motility response relationships (Fig 2A–2C).

### Chemotaxis toward nutrition reproduces the colony morphology difference

Numerical simulations of our model create diverse colony patterns by varying environmental parameters. Among them, the combination of two parameters reproduces the morphological diagram in controlled peptone and pH parameters (Fig 1D and 1E, S1 Text). One is the initial nutrition level *P* regarded as the peptone concentration in the culture experiment. The other is the chemotactic motility coefficient *m*_c_ (Fig 2C); its absence (*m*_c_ = 0) forms the volcano-like pattern (Fig 2D, 2E, 2H and 2J), whereas a positive value (*m*_c_ > 0) the crater-like (Fig 2F, 2G, 2I and 2K–2M). The numerical results support that a slight acid shift from pH 7.4 is associated particularly with chemotactic strength also in collective behavior on solid surfaces (Fig 1F).

### Resource-controlled motility under self-generated resource gradients arranges cell population structures in biofilms

The inverted U-shaped dose-response relationships between nutritional resource level and bacterial motility play a central role in controlling the self-organization of bacterial communities. The resource-response diagram along the log *N* axis (Fig 2C) represents where and how each bacterial activity contributes to colony organization, since resource gradients are generated by consumption by bacterial cells within the expanding colonies and then log *N* shows linear correlation with distance from the inoculation point (Fig 2E and 2G).

According to the resource-response diagram, random motility mainly affects colony growth fronts to expand outer margins and to generate notched outlines (Fig 2E and 2G). Sharp motility increase at intermediate nutrition level (Fig 2C) also corroborates the fast growth of the volcano-like and the crater-like patterns. The inverted U-shaped dose-response curve implies that growing bacteria increase motility as the surrounding nutrient reduces by consumption. When a part of motile bacteria successfully reach a nutritious outer area, they decrease motility and proliferate in new environment to thicken the growth fronts. Earlier nutrient consumption elicits earlier bacterial migration in better environment, so that fluctuations bring an unequal, notched volcano-like outline with thick front margins owing to this controlled positive feedback (Fig 2J–2M). If nutrition falls below a starving line, then bacterial motility declines rapidly to be immotile. They cannot be liberated from nutritional deficiency, which stabilizes the colony outline and prevents adjacent growth fronts from fusing.

Chemotactic motility works effectively in relatively inner regions (Fig 2G). Our model suggests that the presence of the chemotactic motility can transform colony morphology into a crater-like distribution. The chemotactic strength is also controlled by the inverted U-shaped nutrition-motility correlation. The controlled chemotaxis forces cells to migrate toward more favorable places and to halt movement when they arrive at sufficiently nutrient-rich sites, resulting in the crater-like distribution. Simulated velocity fields (Fig 2J–2M) actually indicate that chemotaxis in outermost margins is relatively small (Fig 2M), and also that, just behind the cell-accumulating rim regions, cells are almost immobile (Fig 2K) since outward chemotactic velocity (Fig 2M) cancels out inward diffusional movement (Fig 2L) acting to disrupt the elevated rim. Furthermore, high velocity regions near colony fronts are shallow in the absence of chemotaxis (Fig 2J), whereas deep and followed by rim regions in the presence (Fig 2K). By creating such deep active fronts, chemotaxis helps in filling notched space with the bacteria to bring a circular outline and a precursor of flat inner regions in later stages.

**Fig 3:**
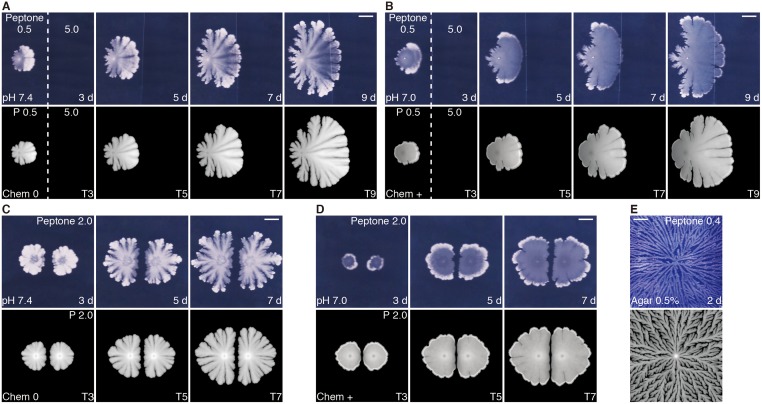
Colony growth simulation in different situations. Experimental (upper) and simulated (lower) patterns. (A, B) Colony growth on inhomogeneous media. Initial nutrition level, 0.5 g l^-1^ peptone (upper), *P* = 0.5 (lower) in left half; 5.0 g l^-1^ peptone (upper), *P* = 5.0 (lower) in right half. (C, D) Mutually interfering two adjacent colonies. Initial nutrition level, 2.0 g l^-1^ peptone (upper); *P* = 2.0 (lower). (E) Another type of colony pattern, dense-branching morphology (DBM)-like, developing in high-moisture and low-nutrient environment (0.5% agar and 0.4 g l^-1^ peptone). Chemotactic coefficients, Chem 0 for pH 7.4 (A, C, E), Chem + for pH 7.0 (B, D). Scale bars, 10 mm.

Motile cells can actually be found in growing colonies by microscopy (S1 and S2 Movies). In both volcano-like and crater-like colonies, most of cells around colony boundaries do not move and form long chains by proliferation without cell separation, while a fraction of cells show motility, swimming around in a local area. The minority of the cell population may assist the expansion of the growth fronts of the colony. Motile cells have a range of distribution. In view of crater-like colonies, moving cells are distributed from colony boundaries to a little inside rim regions (~ 1 mm from the boundaries). In the innermost regions there are no motile cells. Although we have not yet realized the quantification of such motile behaviors, these observations appear to be consistent with the above consideration and the simulated vector fields of motility (Fig 3E, 3G and 3J–3M). No effective motility has been often assumed in this range of hard agar media, but these findings suggest that motile cells play a role in organizing spatio-temporal structures of thick biofilms on solid surfaces.

### Model simulation in different conditions

The model simulation tells us the future microbial population structures even in complex situations. For example, it is applicable to colony growth in deformed environment, such as patched agar plates containing low and high nutrition. If bacteria are inoculated on the lower nutrient side, then their colony expands toward the higher, forming a sea anemone-like (pH 7.4) or jellyfish-like (pH 7.0) outline, which can be predicted by our model (Fig 3A and 3B). Another example is a competitive situation. Multiple adjacent colonies exhibit mutual interference, and our model informs us, for instance, whether they fuse or not, which depends on the culture conditions such as the nutrition level and the positional relationship between the inoculation points (Fig 3C and 3D). Our model can produce another colony pattern, dense-branching morphology (DBM)-like [13, 46, 47], which is observed in high-moisture, low-nutrient environment (Fig 3E). In all the cases, the developmental time series and the environmental parameters are linearly correlated with those in the actual colony culture. Thus, our model appears to be valid and robust in predicting bacterial colony growth.

## Discussion

Our synergistic analysis of biological and mathematical approaches suggests that resource-controlled motility is a key feature for organizing morphologies of the bacterial biofilm. By the capillary aggregation assay we evaluated two kinds of motile property, chemotactic and random migration, and found both were regulated in a manner of inverted U-shaped dose-response along nutrient resource level (Fig 2A–2C). This behavioral appearance indicates the bacterial flagellar activity is highly dependent on surrounding nutrient level, probably due to the survival strategy. They migrate less in nutrient-rich environment whereas increase motility in nutrient-poor to find a better place to live. Below a critical starvation level, they may abandon migration to hibernate to survive. Another feature is suggested from our model in which exponential gradient of nutrient resource is dynamically generated within the expanding biofilms, since the nutrient is consumed by living bacteria through the Michaelis-Menten-like kinetics and is therefore finally exhausted exponentially (Fig 2E and 2G). Thus, resource-controlled motility with self-generated gradient is assumed to produce systematical collective behaviors in the same manner as directed migration of cell collectives in eukaryotes.

In this study we demonstrated small pH alteration in a neutral range induces a morphological change in bacterial biofilms: A volcano-like pattern with a notched outline was formed at pH 7.4, whereas a crater-like pattern with a circular outline and an elevated rim was at pH 7.0. In the capillary assay, chemotactic motility significantly increased in pH 7.0, and our simulation study indicated chemotactic motility tends to fill notched space and to make a circular outline. Chemotaxis also promotes radial migration within the biofilm and forms rim structures on the expanding front. At the moment chemotactic activity was neglected in the simulation for volcano-like patterns formed at pH 7.4, whereas a smaller degree of chemotactic activity, compared with pH 7.0, was still observed in pH 7.4 media in the experiment. Moreover, the motility and chemotactic coefficients for pH 7.0 were chosen to have narrower effective ranges (FWHM) compared with those observed in the capillary assay. As we stated, this disparity probably comes from the conditional difference between the capillary assay examining only motile cells in liquid media and collective motility on hard agar plates. Although pH fluctuation may moderately affect individual bacterial cell chemotaxis in liquid, our results suggest collective motility and chemotaxis are more sensitive to pH on agar plates probably owing to the selection of cell types [48–50]. More detailed analysis of motility including gene regulatory feedbacks on distinct cell states may reveal realistic coefficients on surface environment for a deeper and more quantitative understanding and accurate prediction of the self-organization of microbial communities. This possibility should be studied and evaluated in future studies.

With regard to biological relevance, it remains unclear if and how the change of the morphology brings some advantage for the bacterial biofilm community. Many microbes adapt their colony structures to their surrounding environment for survival and maintenance, by means of fast-expansion of their territory, energy-saving under poor resources, or preventing other species from invasion. Since intracellular pH of *B*. *subtilis* is reported to be pH 7.4 (the volcano-like colony condition) [27], pH 7.0 (the crater-like colony condition) may be recognized as a sign of upcoming acidic stress in environment. In this aspect, the filling of notched space and cell accumulation on the rim would potentially bring protective features against acidic fluctuations in addition to efficient nutrition availability. Further investigation should be required to validate biological relevance of the biofilm morphologies, although the flexible growth design of the biofilm may be more strategic than previously thought. Notwithstanding that more complex behavior should be considered for higher organisms, we can still learn universal principles from rational strategies of microbial systems.

## Materials and methods

### Bacterial strain and preculture

*B*. *subtilis* wild-type strain OG-01 [8] was used. A very small quantity of bacteria (endospores) was incubated overnight (10–16 h) in 5 ml of LB liquid growth medium (LB Broth Base (Lennox L Broth Base), Gibco BRL Life Technologies, cat. no. 12780–052) at 35°C with shaking at 140 rpm (Bio-shaker BR-40LF, Taitec). Approximately 50 μl of bacterial suspension was incubated again in 5 ml of new LB medium for about 3.5 h. The suspension was centrifuged for 5 min at 3000 rpm (GS-6R Centrifuge, Beckman), and the pellet was suspended in 5 ml of ‘wash’ buffer (38 mM NH_3_Cl, 29 mM KH_2_PO_4_, and 40 mM NaH_2_PO_4_; pH 7.0). Then, the suspension was centrifuged again, so that the pellet contained almost no nutrients and a sufficient quantity of bacteria with the highest motility (over 90% of the bacteria were very motile). For inoculation on solid agar media, this final pellet was diluted with the buffer to OD_600_ = 0.5.

### Liquid nutrient media

A solution of 86 mM NaCl and 29 mM K_2_HPO_4_ was first prepared. As a nutrient, a designated amount of peptone (Bacto Peptone, BD Biosciences, cat. no. 211677) was added into the solution, and the pH was then adjusted at a designated value by adding 6 M HCl.

### Solid nutrient agar media

For culture experiments of bacterial colonies, basically, 1% agar (Bacto Agar, BD Biosciences, cat. no. 214010) was added into the above liquid nutrient medium. After autoclaving at 121°C for 15 min, 20 ml of the solution was poured into each plastic petri dish. The dishes were kept at room temperature overnight and dried at 50°C for 30 min. For the half-and-half media (Fig 3a and 3b), 20 ml of one of the two kinds of media, which corresponded to the left half domain including the inoculation point, was first poured into a petri dish. The dishes were kept at room temperature overnight and dried at 50°C for 60 min. The right half of the media were then removed with the help of sterilized knife and spoon. Finally, 10 ml of the other kind of medium was poured into the left half space of the dish. The dishes were kept at room temperature for only 5–10 min to minimize the diffusion of nutrient before the beginning of colony growth.

### Inoculation and incubation

The pre-cultured bacterial suspension was inoculated by using a sterilized needle basically at the center of the prepared solid medium surface. The dishes were incubated in a humidified box at 35°C for designated time. The macroscopic colony images were obtained by using a flat scanner (CanoScan LiDE 110, Canon) and a stereomicroscope (M125, Leica Microsystems) with a camera (DFC 500, Leica Microsystems). The height profile of colonies was measured with a digital microscope (VHX-1000, Keyence) equipped with a universal zoom lens (VH-Z100UR, Keyence).

### Motility assay

The motility changes due to environmental variations were investigated on the basis of a capillary assay [34, 38–41]. The media contained 86 mM NaCl, 29 mM K_2_HPO_4_, 0.1 mM ethylenediaminetetraacetic acid (EDTA), 0.14 mM CaCl_2_, 0.3 mM (NH_4_)_2_SO_4_, and a designated quantity of peptone: *N* g l^-1^ for ‘wash’, ‘pond’, ‘capillary (in random motility assays)’, *N* + 10 g l^-1^ for ‘capillary (in chemotactic motility assays)’. The pH was adjusted at a designated value by adding 6 M HCl. The pond solution was 200 μl containing about 3 × 10^6^ bacteria ml^-1^. The capillary solution was about 0.4 μl. The capillary was made from a 32 mm glass capillary (1 μl Microcaps, Drummond Scientific Company, cat. no. 1-000-0010). The assays were carried out at 35°C for 45 min.

## Supporting information

S1 Movie Microscope observation near the boundary of volcano-like colony at pH 7.4. (MP4)

S2 Movie Microscope observation near the boundary of crater-like colony at pH 7.0. (MP4)

S1 Text Details of the mathematical model and simulation. (PDF)
