# Supplementary material for: Self-organization of bacterial communities against environmental pH variation: Controlled chemotactic motility arranges cell population structures in biofilms
Source: PLoS One. 2017 Mar 2;12(3):e0173195. doi: 10.1371/journal.pone.0173195 (PMC5333884; doi:10.1371/journal.pone.0173195)
Supplement: S1 Text — (PDF) [file pone.0173195.s003.pdf]

Self-Organization of Bacterial Communities against Environmental pH Variation: Controlled Chemotactic Motility Arranges Cell Population Structures in Biofilms

Supporting Information: Details of the mathematical model and simulation

Sohei Tasaki[1,2*], Madoka Nakayama[3], Wataru Shoji[1,4]

[1]Frontier Research Institute for Interdisciplinary Sciences (FRIS), Tohoku University, Sendai, Japan, [2]Graduate School of Science, Tohoku University, Sendai, Japan, [3]Sendai National College of Technology, Natori, Japan, [4]Institute of Development, Aging and Cancer, Tohoku University, Sendai, Japan

Inverted U-shaped dose-response model

The proposed motility response coefficient describes an inverted U-shaped curve on the log N axis: For $X = \ln N$,

$$m\left(\frac{N}{\kappa+N^2}\right)^{\alpha} = M\,\mathrm{sech}^{\alpha}\left(X-X_M\right);\quad M = m\left(\frac{1}{2\sqrt{\kappa}}\right)^{\alpha},\quad X_M = \ln\sqrt{\kappa},\quad \mathrm{HWHM} = \ln\left(2^{1/\alpha}+\sqrt{4^{1/\alpha}-1}\right)$$

where M is the maximum value, X_M the maximum point, and HWHM the half width at half maximum. The following similar form may also be suitable:

$$m\frac{\kappa^{1-\alpha}N^{\alpha}}{\kappa+\kappa^{1-\alpha}N^{2\alpha}} = M\,\mathrm{sech}\left(\frac{X-X_M}{\sigma}\right);\quad M = \frac{m}{2\sqrt{\kappa}},\quad X_M = \ln\sqrt{\kappa},\quad \mathrm{HWHM} = \sigma\ln\left(2+\sqrt{3}\right),\quad \sigma = \frac{1}{\alpha}$$

Under the following assumptions:

- (Random) cell motility takes the first-order ($\alpha_{\mathrm{r}} \sim 1$) form.
- Chemotactic sensitivity follows the Hill equation with Hill coefficient ~ 2 (positively cooperative binding).
- Chemotactic velocity is described by the spatial-gradient of the chemotactic sensitivity multiplied by the cell motility.

we can approximately derive the same form ($\alpha_{\mathrm{c}} = 2$) of the chemotactic motility coefficient:

$$\mathrm{constant}\times m_{\mathrm{r}}\left(\frac{N}{\kappa_{\mathrm{r}}+N^2}\right)^{\alpha_{\mathrm{r}}}\nabla\left(\frac{N^2}{\kappa_{\mathrm{c}}+N^2}\right) \sim m_{\mathrm{c}}\left(\frac{N}{\kappa_{\mathrm{c}}+N^2}\right)^{\alpha_{\mathrm{c}}}\nabla N$$

Population scale model of spatio-temporal growth of bacterial colonies

In our model, we use two functions: the nutrition level $N = N(\boldsymbol{x}, t)$ and the bacterial concentration $B = B(\boldsymbol{x}, t)$, where $(\boldsymbol{x}, t)$ denotes the space and time variables. The nutrition N diffuses in the media and is consumed by bacterial proliferation:

$$\frac{\partial N}{\partial t} = d\nabla^2 N - cp(N,B)$$

where $p = p(N, B)$ denotes the proliferation speed, d and c are the diffusion and consumption rates. The bacteria B move with the velocity $\boldsymbol{u} = \boldsymbol{u}(N, B)$ and proliferate consuming nutrition:

$$\frac{\partial B}{\partial t} + \nabla\cdot\left(B\boldsymbol{u}\right) = p(N,B)$$

For the proliferation $p = p(N, B)$, we take the following form of the Monod type [51]:

$$p(N,B) = p_{\max} \frac{N}{\kappa_N + N} \frac{B}{\kappa_B + B}$$

In addition to the saturation effect in nutritional increase, it represents bounded proliferation caused by spatial restriction on the two-dimensional medium surface. The maximum growth rate is given by $p_{\max}$. The half speed coefficients are denoted by κ_N and κ_B. Finally, the moving velocity vector is described as:

$$\boldsymbol{u}(N,B) = -m_{\mathrm{r}} \left(\frac{N}{\kappa_{\mathrm{r}} + N^2} \right)^{\alpha_{\mathrm{r}}} \Theta \nabla B + m_{\mathrm{c}} \left(\frac{N}{\kappa_{\mathrm{c}} + N^2} \right)^{\alpha_{\mathrm{c}}} \nabla N$$

Simulations

Numerical calculation was performed by the standard explicit Euler method. We used arbitrary units that were matched to the experimental results. The common parameters were as follows: $d = c = 2$, $p_{\max} = 1$, $\kappa_N = 2$, $\kappa_B = 1$, $m_{\mathrm{r}} = 0.04$, $\kappa_{\mathrm{r}} = 0.04$, $\alpha_{\mathrm{r}} = 1$, $\kappa_{\mathrm{c}} = 10^{-5}$, $\alpha_{\mathrm{c}} = 2$. The chemotactic coefficient is $m_{\mathrm{c}} = 0$ (pH 7.4) or 5×10^{-4} (pH 7.0) unless otherwise noted. The random operator Θ was defined by a diagonal matrix. The diagonal components θ_1 and θ_2 were random numbers, taken at each spatio-temporal step, with a symmetric triangular distribution of mean 1 and support [0, 2]. The system size was 400×400 square lattice. The space and time step size was 1 and 0.02, respectively. The initial data were $N(\boldsymbol{x}, 0) = P$, $B(\boldsymbol{x}, 0) = \Sigma_i I_i(\boldsymbol{x})$, where

$$I_i(\boldsymbol{x}) = 0.1 \times \exp\left(-|\boldsymbol{x} - \boldsymbol{x}_i|^2 / 15\right) \times \chi\left(|\boldsymbol{x} - \boldsymbol{x}_i|\right)$$

This function stands for the inoculation at a designated point $\boldsymbol{x}_i$ on the agar medium surface. Here we used function $\chi = \chi(r)$ defined by $\chi(r) = 1$ if $r < 10$ and $\chi(r) = 0$ if $r \geq 10$. This cut-off function becomes important only when the proliferation speed is much higher than the movement speed. Only for the DBM-like pattern on the nutrient-poor and high-moisture medium (Fig 3E), we used the modified proliferation rate (not essential for DBM pattern formation):

$$\tilde{p}(N,B) = p_{\max} \left(\frac{N - \delta}{\kappa_N + N} \right)_+ \frac{B}{\kappa_B + B}$$

This form was taken because the maintenance energy, namely the energy consumed by the upkeep of fundamental bacterial activity, became quite influential in the nutrient-poor situation [52]. Here, the subscript + means the positive part and $\delta(=0.2)$ represents the maintenance cost rate. The parameters were changed: $d = 2$, $c = 0.075$, $p_{\max} = 200$, $\kappa_N = 5$, $\kappa_B = 10$, $m_{\mathrm{r}} = 15$, $\kappa_{\mathrm{r}} = 2.5$, $\alpha_{\mathrm{r}} = 1$, $m_{\mathrm{c}} = 0$. In

particular, bacteria wildly swarm on surface-moisture rich agar media, and then, collective chemotactic behavior is mostly suppressed [53], which is in agreement with the fact that chemotaxis is not necessary for simulating DBM-like pattern formation.

Front aggregation index

For a quantitative understanding of changing colony patterns, we introduced an index of a spatial structure of cell population. The definition and the calculation method are the following.

First, we prepare a cell population distribution map (e.g., Fig 1E; t = T5 (Tm = 220*m) in simulations). For the results of experiments (5days post-inoculation), we perform the correction of the lowest level (the surrounding agar region) and logarithmic filtering [54]. Then, from the distribution map, we clip a slender rectangular region inscribed in the colony outline including cracks if exist (Fig A). Concerning the present result, for instance, for the length $2R$ (R is the radius of the colony), the width is $0.1R$ (~ 2 mm in experiments). To eliminate fluctuations around the inoculation point, we remove the inner rectangular region of the length R and the same width. Then we evaluate the mean cell density M in the outer half region, and the weighted mean cell density M_w in the same region. The weight function is

$$w(r) = \frac{n+1}{2-2^{-n}}\left(\frac{r}{R}\right)^n$$

where r is the distance from the center, n is an index of front aggregation sharpness (The larger the value of n is, the heavier the weight around colony boundaries is. n = 0 means no weighting. Here we take n = 32). Finally we define a front aggregation index as $FAI = M_w / M$. If the colony is circular and the cell distribution is homogeneous, then FAI = 1. Cell accumulation at growth fronts in crater-like colonies increases the value of FAI, and branching in volcano-like colonies decreases FAI due to the notched space around colony boundaries. Therefore, the index FAI can characterize the morphological change between the volcano-like and the crater-like colonies.

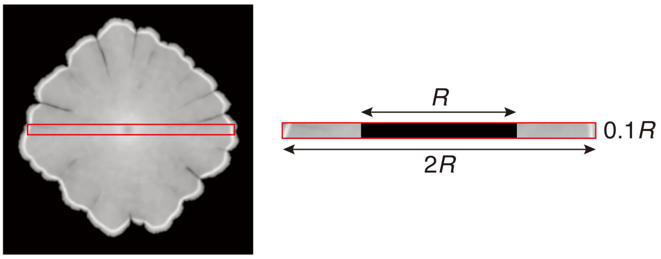

Fig A. Calculation domain for front aggregation index

Supplementary References

51. Monod J. The growth of bacterial cultures. Annu Rev Microbiol. 1949; 3: 371-394.

52. Kovárová-Kovar K, Egli T. Growth kinetics of suspended microbial cells: from single-substrate-controlled growth to mixed-substrate kinetics. Microbiol Mol Biol Rev. 1998; 62: 646-666.

53. Darnton NC, Turner L, Rojevsky S, Berg HC. Dynamics of bacterial swarming. Biophys J. 2010; 98: 2082-2090.

54. Takeuchi R, Tamura T, Nakayashiki T, Tanaka Y, Muto A, Wanner BL, Mori H. Colony-live –a high-throughput method for measuring microbial colony growth kinetics- reveals diverse growth effects of gene knockouts in *Escherichia coli*. BMC Microbiol. 2014; 14: 171.
